# Are there any benefits for post-operative splinting after carpal tunnel release? A systematic review and meta-analysis

**DOI:** 10.1186/s12891-024-07230-6

**Published:** 2024-02-21

**Authors:** Uchenna I. Peter-Okaka, Samira Shiri, Oluwafemi Owodunni, Seyed Reza Bagheri, Amir Jalilian, Cynthia Uzoukwu, Sonia Eden, Ehsan Alimohammadi

**Affiliations:** 1 Department of Neurosurgery, Rutgers University New-Brunswick, Moncton, NJ USA; 2 Clinical Research Development Center, Taleghani and Imam Ali Hospital, Kermanshah University of Medical Sciences, Kermanshah, Iran; 3 Department of Neurosurgery, University of New Mexico Hospital, Albuquerque, NM USA; 4 Department of Neurosurgery, Kermanshah University of Medical Sciences, Kermanshah, Iran; 5 Kermanshah University of Medical Sciences, Kermanshah, Iran; 6 Northeast Ohio Medical University, Rootstown, OH USA; 7 Semmes Murphey Clinic and University of Tennessee Health Science Center, Memphis, TN USA

**Keywords:** Carpal tunnel release, Postoperative care, Splinting, Symptom severity score, Functional status scale

## Abstract

**Background:**

There is a controversy on the effectiveness of post-operating splinting in patients with carpal tunnel release (CTR) surgery. This study aimed to systematically evaluate various outcomes regarding the effectiveness of post-operating splinting in CTR surgery.

**Methods:**

Multiple databases, including PubMed, EMBASE, CINAHL, Web of Science, and Cochrane, were searched for terms related to carpal tunnel syndrome. A total of eight studies involving 596 patients were included in this meta-analysis. The quality of studies was evaluated, and their risk of bias was calculated using the methodological index for non‐randomized studies (MINORS) and Cochrane’s collaboration tool for assessing the risk of bias in randomized controlled trials. Data including the visual analogue scale (VAS), pinch strength, grip strength, two-point discrimination, symptom severity score (SSS), and functional status scale (FSS) were extracted.

**Results:**

Our analysis showed no significant differences between the splinted and non-splinted groups based on the VAS, SSS, FSS, grip strength, pinch strength, and two-point discrimination. The calculated values of the standardized mean difference (SMD) or the weighted mean difference (WMD) and a 95% confidence interval (CI) for different variables were as follows: VAS [SMD = 0.004, 95% CI (-0.214, 0.222)], pinch strength [WMD = 1.061, 95% CI (-0.559, 2.681)], grip strength [SMD = 0.178, 95% CI (-0.014, 0.369)], SSS [WMD = 0.026, 95% CI (- 0.191, 0.242)], FSS [SMD = 0.089, 95% CI (-0.092, 0.269)], and the two-point discrimination [SMD = 0.557, 95% CI (-0.140, 1.253)].

**Conclusions:**

Our findings revealed no statistically significant differences between the splinted and non-splinted groups in terms of the VAS, SSS, FSS, grip strength, pinch strength, and two-point discrimination. These results indicate that there is no substantial evidence supporting a significant advantage of post-operative splinting after CTR.

## Introduction

Carpal tunnel syndrome (CTS) is a common condition characterized by compression of the median nerve as it passes through the carpal tunnel in the wrist [1, 2]. Surgical intervention, known as carpal tunnel release (CTR), is often recommended for moderate to severe cases that do not respond to conservative treatments. Following CTR, there has been a long-standing debate regarding the benefits of postoperative splinting [3–5].

Postoperative splinting involves immobilizing the wrist and hand in a neutral position using a splint or brace after CTR. The rationale behind splinting is to provide support, reduce edema and pain, and promote healing of the surgical site. However, the use of splints after CTR has been a subject of controversy among healthcare professionals [6–8].

Proponents of postoperative splinting argue that it helps to maintain the alignment of the wrist and hand, minimizing stress on the healing tissues and preventing excessive motion that could impede recovery. They believe that splinting can aid in reducing postoperative pain, swelling, and scar formation, leading to improved functional outcomes and patient satisfaction [9, 10].

On the other hand, opponents of splinting argue that it may restrict hand function and delay the recovery process. They suggest that early mobilization of the hand and fingers after CTR may promote better blood circulation, prevent joint stiffness, and facilitate a faster return to normal activities. Additionally, concerns have been raised about the potential for muscle atrophy and decreased grip strength associated with prolonged splint use [10–13].

Given the conflicting viewpoints regarding the benefits of postoperative splinting after CTR, a comprehensive evaluation of the available evidence is necessary to better inform clinical practice. In this meta-analysis, we aim to systematically review and synthesize the existing literature on the controversies surrounding postoperative splinting after CTR. By critically analyzing relevant studies, we seek to provide a quantitative synthesis and evidence-based assessment of the advantages and disadvantages of splinting in terms of pain relief and functional outcomes.

The findings from this meta-analysis will help healthcare professionals make informed decisions regarding postoperative splinting after CTR, ultimately improving patient care and optimizing outcomes for individuals with carpal tunnel syndrome.

## Methods

**Fig. 1:**
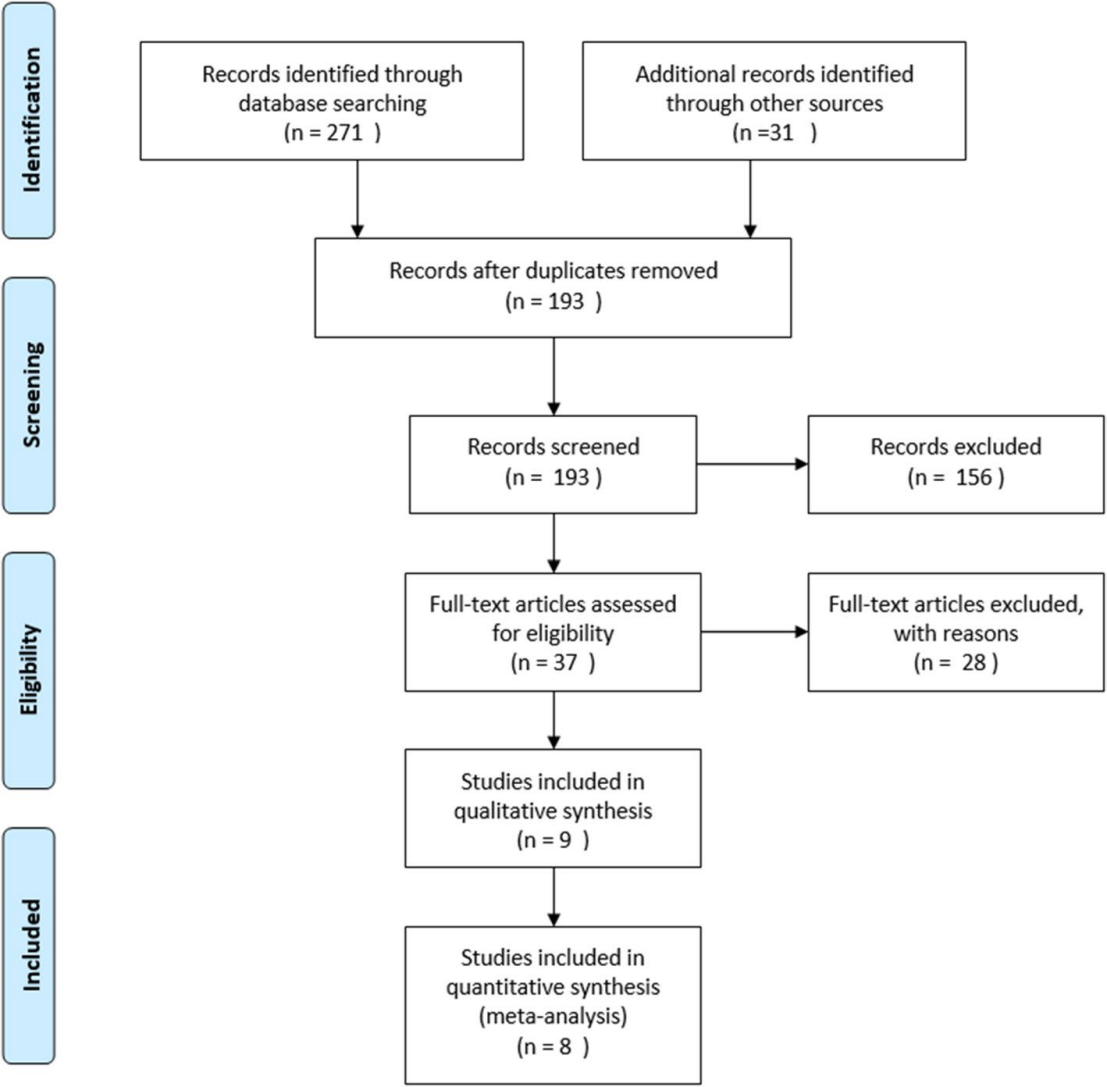
PRISMA flow-diagram showing summary of literature review

The present study was conducted according to the Preferred Reporting Items for Systematic Reviews and Meta-Analyses (PRISMA) statement (Fig. 1) [14].

### Inclusion and exclusion criteria

The inclusion criteria of the study were as follows: 1. Randomized controlled trials (RCTs) and non-randomized controlled trials (non-RCTs), including prospective and retrospective studies. that investigate the use of postoperative splinting after CTR.

2. Studies that report outcome measures related to pain relief and functional outcomes after CTR.

3. Studies available in English.

Case reports, commentaries, editorials, and non-English studies were excluded.

### Study identification

Two independent reviewers (SSh and AJ) performed a comprehensive web-based literature search using PubMed, EMBASE, CINAHL, Web of Science and Cochrane. No date limitations were applied. The following search terms were used:

((("Carpal Tunnel Syndrome"[Mesh]) OR ("Carpal Tunnel Syndrome"[Title/Abstract]) AND (("Carpal Tunnel Release"[Mesh]) OR ("Carpal Tunnel Release"[Title/Abstract]) OR ("Carpal Tunnel Surgery"[Mesh]) OR ("Carpal Tunnel Surgery"[Title/Abstract]))) AND (("Splints"[Mesh]) OR ("Splints"[Title/Abstract]) OR ("Braces"[Mesh]) OR ("Braces"[Title/Abstract]) OR ("Immobilization"[Mesh]) OR ("Immobilization"[Title/Abstract])) AND (("Postoperative Care"[Mesh]) OR ("Postoperative Care"[Title/Abstract])). The literature search was conducted to include studies published from January 1990 to January 2023.

Both authors independently reviewed the titles, abstracts, and full-text studies according to pre-established criteria. Discrepancies were resolved by consensus and a third reviewer being consulted in case of disagreement. We used the weighted kappa scores to evaluate agreement between two researchers [15]. There was a perfect agreement between the two reviewers (κ = 0.87).

### Risk of bias assessments and evaluations of validity

Two independent reviewers (AJ and SSh) evaluated the quality of studies and their risk of bias using the methodological index for non‐randomized studies (MINORS) and the Cochrane’s collaboration tool for assessing risk of bias in randomized controlled trials [16–18]. We determined the high risk of bias using a risk of bias score for non-randomized studies as ≤ 8 (controlled group not present) or ≤ 12 (controlled group present). The risk of bias for randomized controlled trials was classified as unclear bias, low risk of bias, or high risk of bias.

### Data extraction and outcome assessment

The following data were extracted from all eligible studies: first author, year of publication, number of patients, gender, age, study design, visual analogue scale (VAS), the pinch strength, the grip strength, two-point discrimination, symptom severity score (SSS), and functional status scale (FSS).

### Heterogeneity assessments

The I2 statistic and the *P*-value for heterogeneity was used to evaluate the heterogeneity between studies [19]. Substantial heterogeneity was considered as ≥ 50% [16].

### Data analysis and statistical analysis

The STATA meta-analysis software was used to perform data synthesis. The standardized mean difference (SMD) with 95% confidence interval (CI) was used to present the result of data synthesis. We used the random effects model to calculate the results of studies with substantial heterogeneity. The fixed effects model was used to assess the results of studies with low heterogeneity.

## Results

**Fig. 2:**
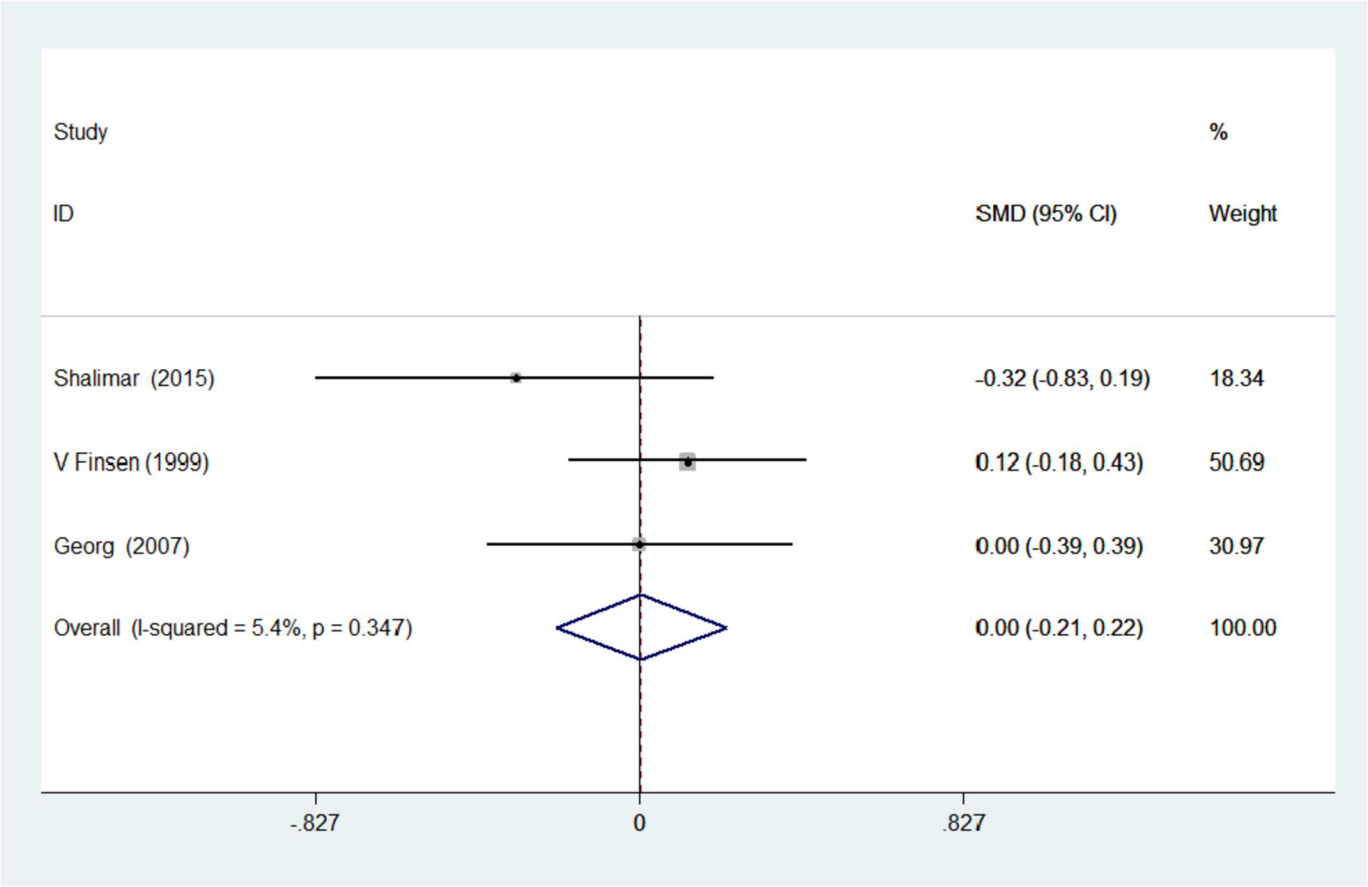
Forest plot of standardized mean difference (SMD) of the four included studies investigating VAS between the splinted and the non-splinted groups

A total of eight studies involving 596 patients were included in this meta-analysis. According to our analysis, there were no significant differences between the splinted group and non-splinted group based on the VAS, SSS, FSS, The grip strength, the pinch strength, and two-point discrimination. Table 1 is summary of evaluated outcomes in all analyzed articles. The VAS was reported in three studies, according to the homogeneity of the studies, I-squared = 5.4%. Their combination was done using the fixed effects model to obtain a Standardized mean difference (SMD) = 0.004 and a 95% confidence interval (-0.214, 0.222) (Fig. 2).

**Table 1:** Summary of evaluated parameters between different groups in all analyzed articles

Article	Number of patients	Regime	Follow-up	Outcome measure(s)
Bury et al. 1995 [4]	26 patients splinted 17 patients non-splinted	Splinted group for 2 weeks Non-splinted group with a bulky dressing	Average 6 months (range, 3.8–7.8 months)	**Grip strength** 26.1 kg strength—splinted 29.4 kg strength—non-splinted. *P* > 0.05 **Pinch strength** 3.9 splinted 3.8 non-splinted **Average subjective score** 8.1. splinted 8.0. non-splinted
Cook et al. 1995 [5]	25 patients splinted; 25 patients non-splinted	Splinted group, volar plaster for 2 weeks Non-splinted group, bulky dressing one day then mobilize as able	2, 4, 12 and 24 weeks	**Grip strength** at 1 month 15 kg strength; splinted 10 kg strength; non-splinted *P* > 0.05 **Pinch strength** Splinted 2 weeks 4 kg; 1 month 5 kg; *P* = 0.01 Non-splinted 2 weeks 6 kg; 1 month 7 kg; *P* = 0.01 **Visual analogue score** Splinted 2 weeks 2.4; 1 month 1.5; *P* = 0.01 Non-splinted 2 weeks 0.9; 1 month 0.5; *P* = 0.01
Finsen et al. 1999 [13]	37 patients splinted 45 patients non-splinted	All patients, bulky dressing for 2 days Splinted group, POP with wrist in dorsiflexion for 4 weeks Non-splinted group, light dressings with free movement	6 weeks and 6 months	**Grip strength** 6 weeks Splinted 76 (71–85); Non-splinted 78 (70–86); *P* > 0.05 **Visual analogue scale (0–100)** Splinted Pre-op 56 2 weeks 6 6 weeks 6 6 months 3 Non-splinted Pre-op 51 2 weeks 5 6 weeks 2 6 months 2
Martins et al. 2006 [7]	26 patients splinted 26 patients non-splinted	All patients in soft dressing and light compressive bandage for 48 h Splinted group, in neutral position for 2 weeks Non-splinted, no further immobilization	2 weeks	**Symptom severity (component of Boston score)** Splinted: Pre-op 33.38 (7.33) vs. post-op 11.38 (4.57) Non-splinted: Pre-op 31.77 (7.56) vs. post-op 12.33 (4.77) **2-point discrimination index** Splinted: 5.85(2.8) pre-op vs. 3.69(1.19) post-op Non-splinted: 7.92 (3.12) pre-op vs. 5.12(2.53) post-op. P > 0.05
Cebesoy et al. 2007 [8]	20 patients splinted 20 patients non-splinted	Patients randomized to splints or bulky dressings post-op for 10 days	1- and 3-month follow-up	**Symptom severity (component of Boston score)** Splinted Pre-op 37.75 1 month 16.50 3 months 13.50 *P* < 0.001 non-splinted Pre-op 36.32 1 month 16.84 3 months 13.50 11.90 *P* < 0.001 **Functional status (component of Boston score)** Splinted Pre-op 26.60 1 month 13.50 3 months 10.65 *P* < 0.001 non-splinted Pre-op 26.11 1 month 12.90 3 months 10.26 *P* < 0.001
Huemer et. al 2007 [10]	25 patients splinted 25 patients non-splinted	Patients were randomized to receive either a light bandage or a bulky dressing with a volar splint left in place for 48 h with the wrist in a neutral position	2 days, 3 months	**Grip strength (mean)** Splinted group Preoperative 50 kg 3-month follow-up 44 kg Non-splinted group Preoperative 47 kg 3-month follow-up 40 kg **Pain (VAS, mean)** Splinted group Preoperative 5 Postoperative day 2 2 3-month follow-up 1 Non-splinted group Preoperative 4 Postoperative day 2 2 3-month follow-up 1 **Two-point discrimination (mean)** Splinted group Preoperative 7 mm 3-month follow-up 6 mm Non-splinted group Preoperative 7 mm 3-month follow-up 6 mm **Scar tenderness** Splinted Group No pain 16 Pain with pressure 7 Pain at rest 2 Non splinted group No pain 16 Pain with pressure 9 Pain at rest 0 **Pick-up test (mean)** Splinted group Pick-up test (mean) Preoperative 21 s 3-month follow-up 19 s Non-splinted group Preoperative 19 s 3-month follow-up 17 s **Distal motor latency** **(improvement; mean)** Splinted group 3-month follow-up 2.47ms Non-splinted group 3-month follow-up 2.48ms
Shalimar et. Al 2015 [3]	16 hands splinted, 14 hands non-splinted	One group was applied with a volar plaster splint (8 layers of Gypsona ®) for one week with the wrist in a 15-degree extended position and full finger motion allowed. The second group had a soft bulky dressing applied for a week with unrestricted active motion	1 week, 2 months, 6 months	**VAS** Splinted immediate– 4.7 1 week – 2.6 2 month – 1.3 6 month – 0.1 Non-splinted Immediate– 5.6 1 week – 2.6 2 month – 1.4 6 month – 0.4 **2-point discrimination (radial, ulnar)** Splinted preop– 7.0,5.8 1 week – 5.3,5.6 2 month – 5.0,4.8 6 month – 4.3,4.3 Non-splinted preop– 6.4,6.2 1 week – 5.7,6.2 2 month – 5.6,5.6 6 month – 4.7,5.3 **Pinch strength** Splinted preop– 4.9 2 month – 4.3 6 month – 6.0 Non-splinted preop– 4.5 2 month – 5.1 6 month – 6.2 **Grip strength** Splinted preop– 18.4 2 month – 16.3 6 month – 21.4 Non-splinted preop– 18.9 2 month – 18.5 6 month – 22.9 **APB** Splinted preop– 3.9 2 month – 3.8 6 month – 4.5 Non-splinted preop– 3.7 2 month – 3.5 6 month – 4.4 **Boston Score (part I, part II)** Splinted preop– 2.6, 2.9 1 week – 1.6 2 month – 1.2, 1.5 6 month – 1.0, 1.0 Non-splinted preop– 2.5, 2.8 1 week – 1.9 2 month – 1.4, 1.4 6 month – 1.1, 1.0 (*p* value for comparison of each value ranged from 0.07 to 0.98)
Logli et al. 2018 [9]	80 no splint, 83 removable splint, 86 nonremovable splint	no-orthosis group—soft dressing with gauze and replacement with adhesive bandage on day 5 Removable-orthosis group—Wrist Brace worn at night over soft dressing. Wrist fixed in a 20-degree extended position Plaster nonremovable-orthosis group – cotton wrap over gauze with plaster orthosis across the volar wrist and molded to keep the wrist in approximately 20 degrees of extension and allowing full digital range of motion	10–14 days, 6 weeks, 3 months, 6 months, 12 months	**Symptom Severity Scale (Mean)** No orthosis 10–14 days– 1.84 6 week – 1.83 3 months – 1.56 6 months – 1.43 12 months – 1.51 Removable orthosis 10–14 days– 1.88 6 week – 1.88 3 months – 1.42 6 months – 1.44 12 months – 1.32 Nonremovable orthosis 10–14 days– 1.87 6 week – 1.90 3 months – 1.62 6 months – 1.41 12 months – 1.46 **Functional Severity Scale (Mean)** No orthosis 10–14 days– 2.30 6 week – 1.67 3 months – 1.45 6 months – 1.40 12 months – 1.34 Removable orthosis 10–14 days– 2.27 6 week – 1.63 3 months – 1.40 6 months – 1.23 12 months – 1.27 Nonremovable orthosis 10–14 days– 2.56 6 week – 1.84 3 months – 1.59 6 months – 1.44 12 months – 1.37 **QuickDASH (Mean)** No orthosis 10–14 days– 42.4 6 week – 26.4 3 months – 19.4 6 months – 11.8 12 months – 11.8 Removable orthosis 10–14 days– 40.6 6 week – 21.8 3 months – 14.5 6 months – 10.6 12 months – 9.1 Nonremovable orthosis 10–14 days– 48.3 6 week – 27.1 3 months – 18.4 6 months – 14.4 12 months – 14.3 **NPRS Pain at Rest (Mean)** No orthosis 10–14 days– 1.58 6 week – 1.61 3 months – 0.85 6 months – 0.46 12 months – 0.56 Removable orthosis 10–14 days– 1.47 6 week – 1.60 3 months – 0.70 6 months – 0.59 12 months – 0.71 Nonremovable orthosis 10–14 days– 1.50 6 week – 1.43 3 months – 0.36 6 months – 0.28 12 months – 0.50 **NPRS Pain in action (Mean)** No orthosis 10–14 days– 2.88 6 week – 3.00 3 months – 2.85 6 months – 1.44 12 months – 1.56 Removable orthosis 10–14 days– 2.40 6 week – 2.27 3 months – 2.11 6 months – 1.60 12 months – 1.29 Nonremovable orthosis 10–14 days– 2.40 6 week – 2.12 3 months – 2.64 6 months – 2.00 12 months – 1.54

*VAS* Visual analogue score, *APB power* Medical research muscle power grade of the Abductor pollicis brevis, *NPRS* Numeric pain rating scale, *QuickDASH* shortened version of the Disabilities of the Arm, Shoulder and Hand (DASH) outcome measure

**Fig. 3:**
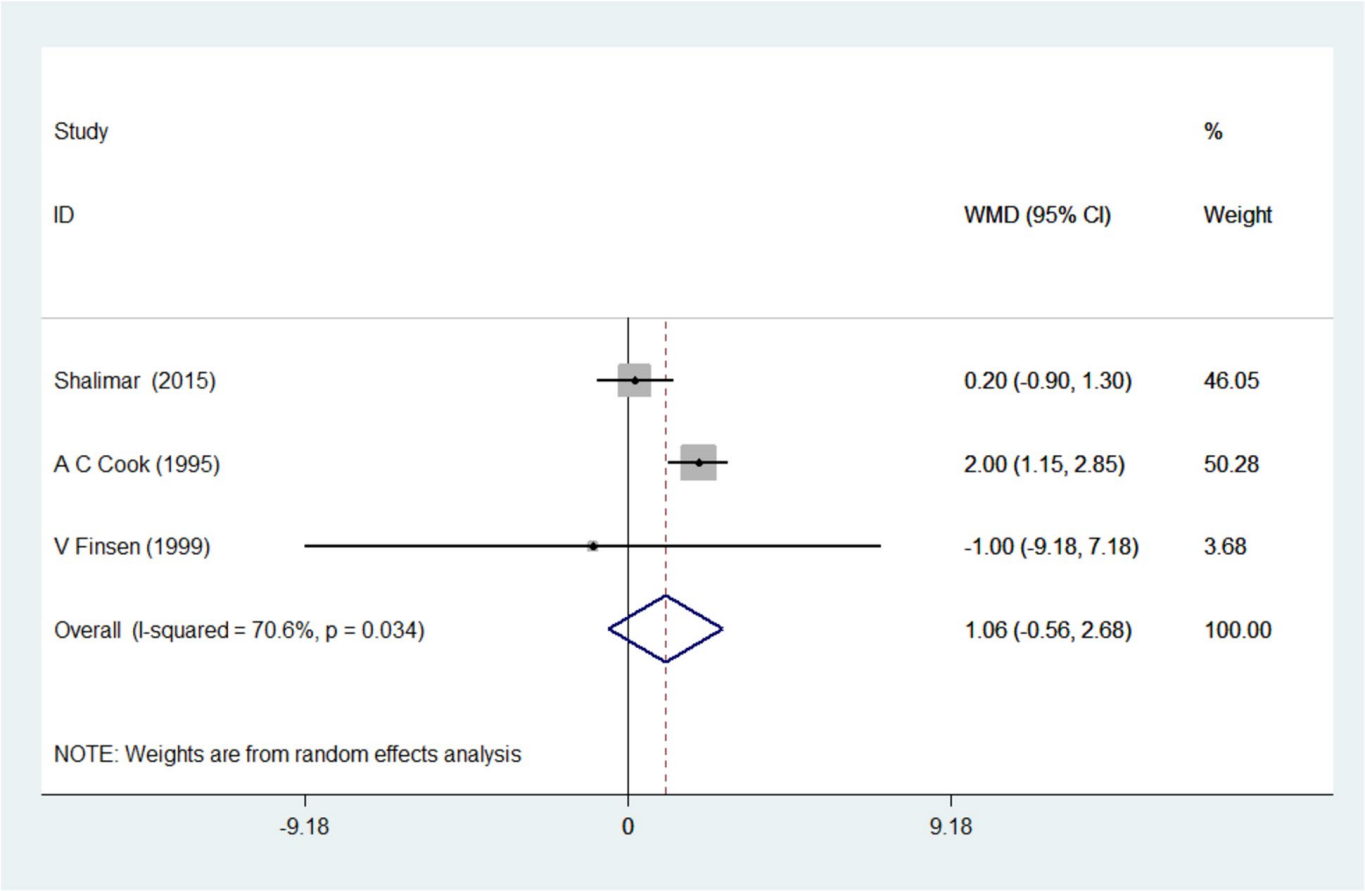
Forest plot of weighted mean difference (WMD) of the three included studies investigating pinch strength between the splinted and the non-splinted groups

The pinch strength was reported in 3 studies and after accounting for the heterogeneity of the studies, I-squared = 70.6% was obtained. Their combination was done using the random effects model to obtain a Weighted mean difference (WMD) = 1.061 with a 95% confidence interval (- 0.559, 2.681) (Fig. 3). Meanwhile, the mean and standard deviation (SD) of pinch strength before and after CTR for the splinted and non-splinted groups (Kg) were as follows: splinted group (before CTR: 6.4 ± 1.9, after CTR: 7.8 ± 2.0) and non-splinted group (before CTR: 6.6 ± 1.8, after CTR: 8.0 ± 2.1).

**Fig. 4:**
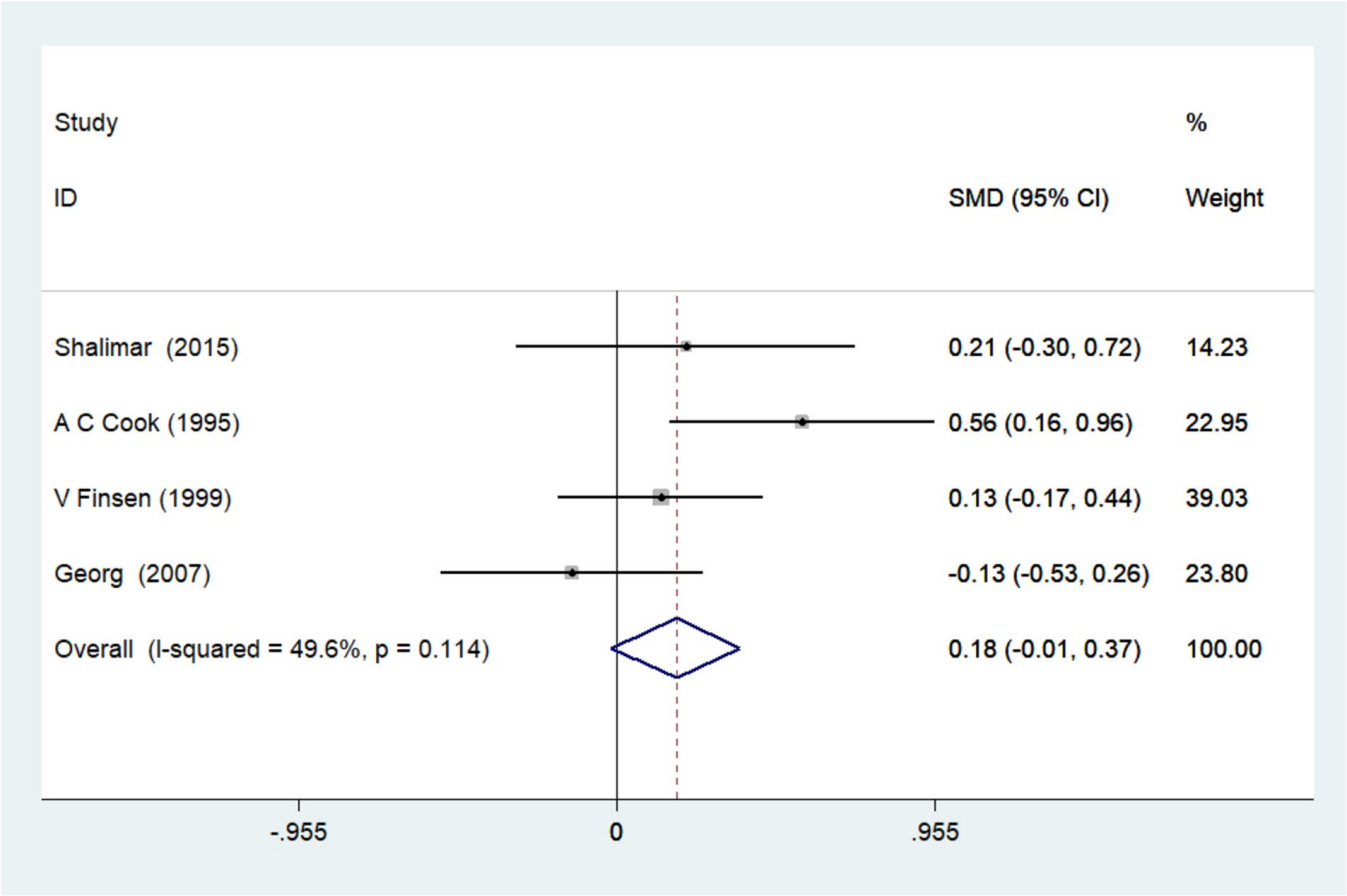
Forest plot of standardized mean difference (SMD) of the four included studies investigating grip strength between the splinted and the non-splinted groups

The grip strength was reported in four studies and due to the homogeneity of the studies, I-squared = 49.6% was obtained. Using a fixed effects model for their combination, SMD = 0.178 with a 95% confidence interval (-0.014, 0.369) was obtained (Fig. 4).

**Fig. 5:**
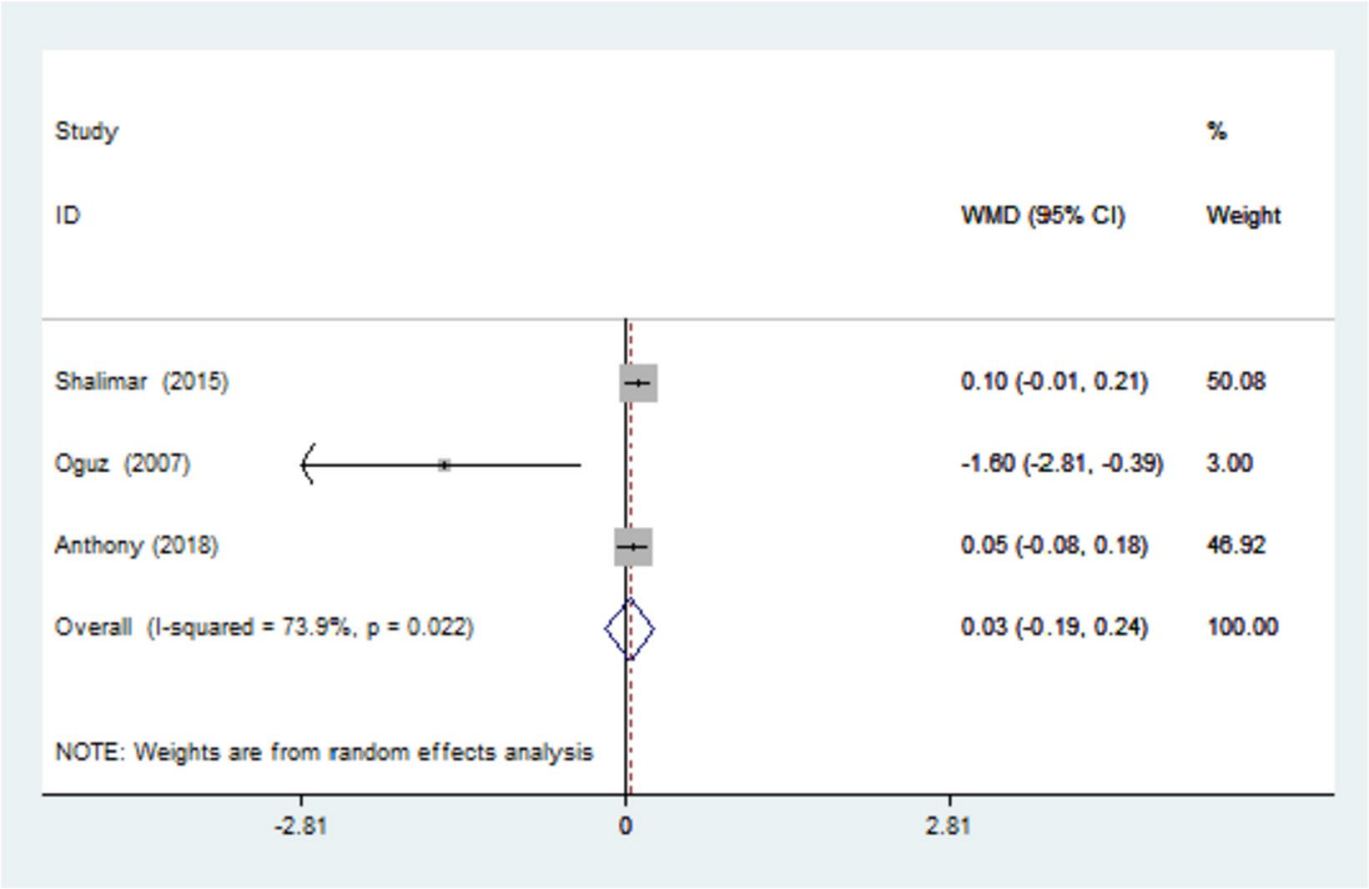
Forest plot of weighted mean difference (WMD) of the three included studies investigating SSS between the splinted and the non-splinted groups

The symptom severity scale (SSS) was reported in three studies with an I-squared = 73.9% due to the heterogeneity of the studies. Their combination was done using the random effects model. (WMD = 0.026) with a 95% confidence interval (- 0.191, 0.242) (Fig. 5).

**Fig. 6:**
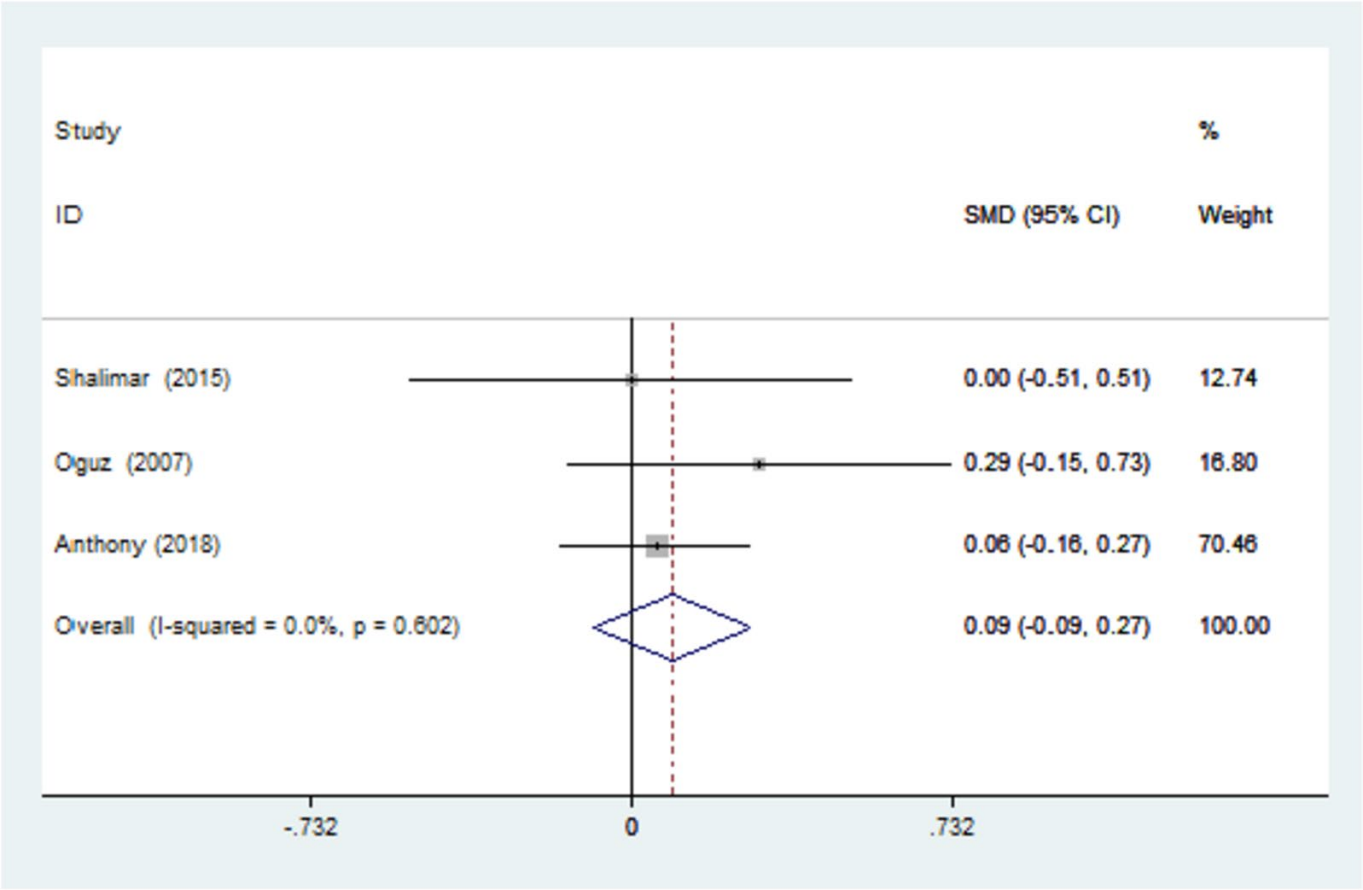
Forest plot of standardized mean difference (SMD) of the three included studies investigating FSS between the splinted and the non-splinted groups

The functional status scale (FSS) was reported in 3 studies and due to the homogeneity of the studies, I-squared = 0.0% was obtained. Their combination was done using the fixed effects model with SMD = 0.089 and a 95% confidence interval (-0.092, 0.269) (Fig. 6).

**Fig. 7:**
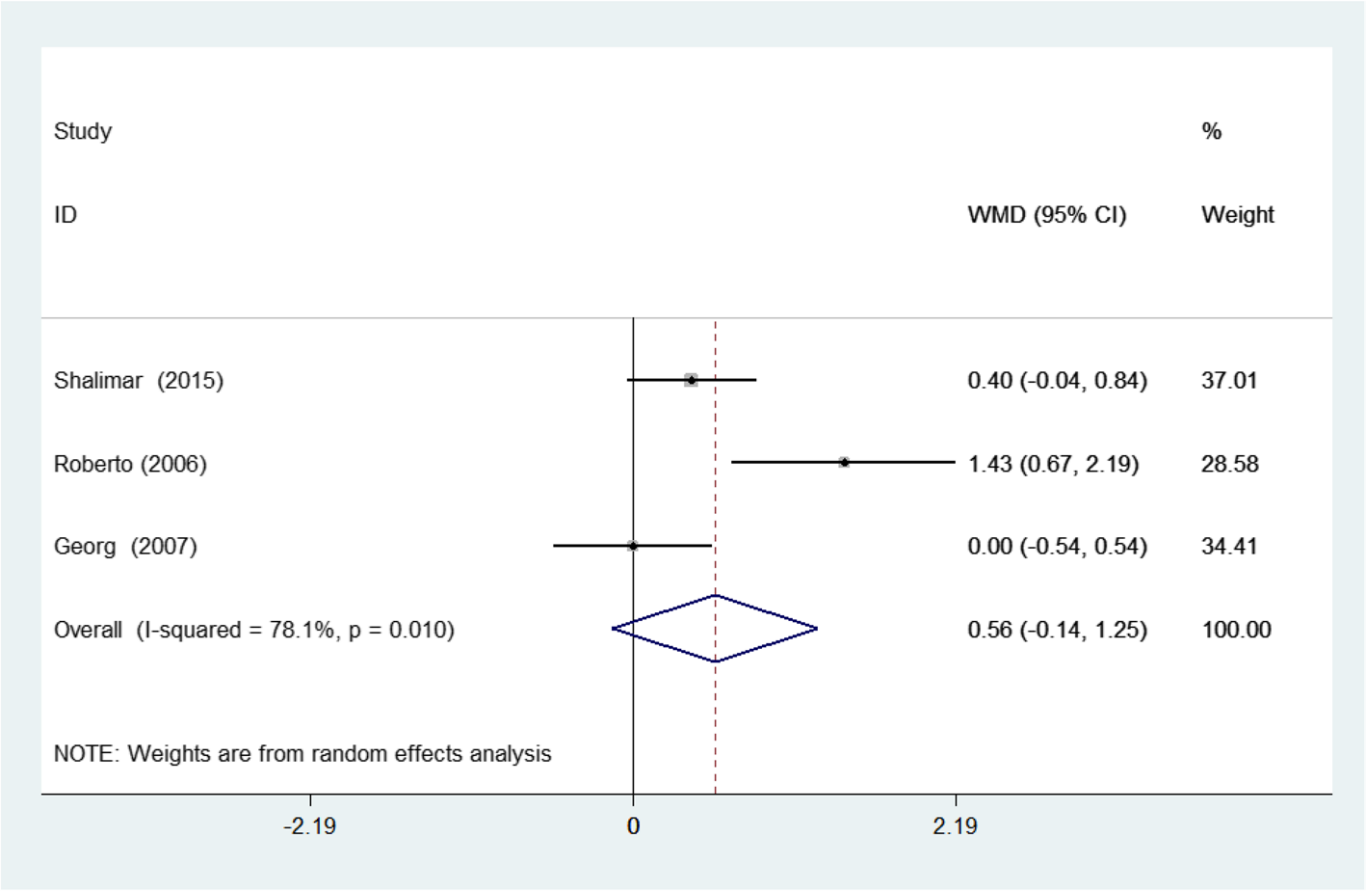
Forest plot of weighted mean difference (WMD) of the three included studies investigating two-points discrimination between the splinted and the non-splinted groups

The two-point discrimination was reported in three studies. Due to the heterogeneity of the studies, I-squared = 78.1%, Their combination was done using the random effects model with WMD = 0.557 and a 95% confidence interval (-0.140, 1.253) (Fig. 7).

No publication bias was identified.

## Discussion

This meta-analysis supports findings from published data which have shown that when compared to the non-splinted group, splinting does not lead to improved outcomes after CTR. The visual analogue score, a measure of pain following CTR was evaluated following an analysis of the studies by Shalimar et al., Huemer et al. and Finsen et al. with a SMD of 0.004 95% C.I. (-0.214, 0.222) (*P* = 0.347), it appears that surgeons do not have sufficient evidence to recommend splinting as a way of reducing post-operative pain [10]. Shalimar et al. analyzed 46 patients divided into the splinted and non-splinted groups with the eventual discovery that post-operative immobilization with a splint did not produce significant benefits with regard to the prevalence of pillar pain postoperatively at one week, two months, and six months (*P* =  > 0.05) [3]. Instead, they found that bulky dressings and splints only contributed to the overall cost at the expense of the patient’s comfort following surgery [3]. Heumer et al. discovered that even after all patients in both study splinted and non-splinted study cohorts had received non-steroidal anti-inflammatory agents for pain control, there was no significant difference in request for the medication between each group, an indirect measure of their level of pain after the procedure on the 2nd postoperative day [10]. This observation also extended to reports of scar tenderness, an issue experienced by two out of fifty patients. Finsen et al. reported that complaints of pain, tenderness, and dysesthesia in the scar were present in both patient cohorts in an evenly-distributed manner and continued to persist at 6 weeks as well as after 6 months [13]. They concluded that immobilization plays little or no role in reducing the frequency of scar pain. These observations are reconfirmed by findings which have been published herein.

Furthermore, the pinch strength and grip strength are important parameters that must be considered following CTR surgery as it is a common indicator of normal return to function of regions supplied by the dermatome of the median nerve and its branches. Measuring the grip and pinch strength can be a reliable way of assessing the intrinsic and extrinsic muscles especially if there is a way to measure the force and torque produced by each muscle. For this reason, dynamometers are usually employed and have been found to have significant reliability and validity [20]. Three studies by Shalimar et al., Cook et al., and Finsen et al. were combined for an analysis of the pinch strength [3, 5, 13]. The substantial heterogeneity of the studies (I-squared = 70.6%) prompted combination using a random effects model to obtain WMD = 1.061, 95% confidence interval (0.559, 2.681). While Shalimar et al. reported a significant increase in pinch strength (*p* = 0.042) in the splinted group at one week, two months and six weeks post-operatively, Finsen et al. found no significant difference between the two groups after evaluation at six weeks and six months postoperatively [3, 13]. However, the findings of Cook et al. seemed to contradict those of Shalimar et al.; they reported that after two weeks, the non-splinted group recovered more rapidly during the assessment of pinch strength (*P* = 0.01) and continued to be observed after 1 month (*P* = 0.01) but did not differ by the third month.

A similar trend was also noted for the grip strength in the three studies reported above, however a total of four studies was evaluated for this parameter. Thus, Heumer et al. additionally confirmed that no significant difference could be found between the splinted and non-splinted groups in the assessment of grip strength [10]. It should be noted that less heterogeneity was noted for the grip strength (I-squared = 49.6%) prompting a combination using a fixed effects model (SMD = 1.78, 95% C.I. (-0.014, 0.369).

The SSS and FSS were reported in three studies and followed a trend identical to the pinch and grip strength regarding the heterogeneity and homogeneity respectively. The forest plots further demonstrate with the results reported that there was no significant difference in improvement regarding these two measures in both splinted and non-splinted cohorts. This is an unsurprising finding following similar reports in previous studies. The Boston questionnaire, a disease-specific questionnaire with two parts was utilized by all three studies [3, 6, 8, 9]. Logli et al. assessed the SSS and FSS up to one year postoperatively and continued to find no significant difference in outcome [9]. While there is currently no plausible explanation for the heterogeneity, we recommend that surgeons should be wary of recommending splinting to patients in a bid to decrease the severity of their symptoms or in an attempt to improve their functional status. It should also be noted that not all of the studies accounted for confounders such as commencement of physical therapy. Cook et al. had previously postulated that early initiation of physical therapy regimens such as exercise postoperatively yields better results in non-splinted patients [5]. Indeed Cebesoy et al. found a significant difference in the third month for the SSS but not for the FSS which was attributed to application of immediate rehabilitation in the non-splinted group but that contrarily, patients in 80% of patients in the splinted group experienced more discomfort attributed to the splint [8].

Studies by Martins et al., Huemer et al. and Shalimar et al. were analyzed for a determination of two-point discrimination [3, 7, 10]. The wide heterogeneity, confirmed by an I-squared of 78.1%, SMD = 0.557, 95% C.I. (-0.140, 1.253) could be explained by the suspected variability in measurements among study groups. Only Martins et al. reported the exact method used for evaluating the two-point discrimination while the other two studies failed to do this. Martins et al. reported that static two-point discrimination was measured using a two-point discriminator (North Coast Medical Inc., California, USA) applied to palmar surface of the second finger distal phalange [7]. Generally, an increase in two-point discrimination was observed between the splinted and non-splinted groups but there was no statistically significant difference between the two groups. This finding implies that surgeons do not need to recommend splinting for patients with the belief that it could help with outcomes such as two-point discrimination since no significant difference has been observed. Additionally, the cost of splinting in addition to the discomfort precludes such a recommendation.

### Limitations

This meta-analysis is limited by several factors. Acknowledging heterogeneity and studies with a high risk of bias as limitations in the synthesis is crucial for providing a comprehensive and transparent assessment of the validity and generalizability of the findings. The presence of moderate heterogeneity among the included studies can introduce variability in the results and limit the overall strength of the conclusions. Heterogeneity may stem from differences in study design, patient populations, surgical techniques, outcome measures, or other factors. Acknowledging this limitation highlights the need for caution when interpreting the pooled results and emphasizes the importance of considering the context and characteristics of individual studies.

Another limitation to consider is the inclusion of studies with a high risk of bias. The risk of bias assessment evaluates the methodological quality and potential biases in individual studies, including issues such as selection bias, performance bias, detection bias, attrition bias, and reporting bias. Studies with a high risk of bias may introduce systematic errors that can affect the reliability and validity of the synthesized results.

Treatment effect heterogeneity poses a significant risk for error. While the included studies shared a common focus on postoperative splinting after CTR, there may be variations in study populations, surgical factors, and rehabilitation protocols that could contribute to the observed heterogeneity.

One possible reason for heterogeneity could be differences in the characteristics of the study populations. Factors such as age, gender distribution, severity of carpal tunnel syndrome, and comorbidities among the participants may vary across studies. These differences in patient profiles could introduce variability in outcomes related to postoperative splinting.

Surgical factors, including variations in the surgical technique, surgeon experience, and use of different types of splints or braces, may also contribute to heterogeneity. These factors can influence the biomechanical forces applied to the wrist and hand during the postoperative period, potentially impacting the effectiveness of splinting in promoting recovery.

Furthermore, variations in rehabilitation protocols, including the duration and intensity of splint use, as well as the timing and type of hand therapy interventions, may contribute to heterogeneity. Differences in the duration of follow-up assessments across studies could also affect outcome measurements.

To better understand the sources of heterogeneity, future research or subgroup analyses within the meta-analysis could be conducted to explore the impact of these factors. By examining the influence of patient characteristics, surgical factors, and rehabilitation protocols, a more comprehensive understanding of the variations in study outcomes can be achieved, helping to inform clinical practice and guide recommendations regarding postoperative splinting after CTR.

On the other hand, while the I-squared statistic has been advertised as a reliable measure to quantify the effect of heterogeneity, it remains a challenge to describe the exact effect of a treatment effect heterogeneity when a mixed model of analysis is indicated.^21^ Using a priori definitions, we identified cut-off points used for assessment of heterogeneity and utilized either a fixed effects or random effects model based on the value of I-squared. Furthermore, only eight studies were used in the meta-analysis. While statistical analyses methodology takes into account various challenges that are commonly encountered while working with such a small number of studies, a greater number of studies would have been more beneficial for our purpose.

Expanding on the clinical implications and recommendations based on our findings, it is important to consider a more selective approach to postoperative splinting after carpal tunnel release. Our study suggests that there may not be a significant advantage to routine prolonged splinting for all patients. Therefore, it is prudent to recommend selective or short-term splint use where indicated, taking into account individual patient factors such as the severity of symptoms, postoperative discomfort, and functional status. This personalized approach aligns with the evolving trend toward tailored interventions in rehabilitation and orthopedic care, emphasizing the need to optimize patient outcomes through individualized treatment plans.

## Conclusions

Our findings revealed no statistically significant differences between the splinted and non-splinted groups in terms of the VAS, SSS, FSS, grip strength, pinch strength, and two-point discrimination. These results indicate that there is no substantial evidence supporting a significant advantage of post-operative splinting after CTR.

## Data Availability

All data generated or analyzed during this study are included in this published. article.
